# Genotoxicity Assessment of Silver Nanoparticles Produced via HVAD: Examination of Sister Chromatid Exchanges in *Chinchilla lanigera* Blood Lymphocytes In Vitro

**DOI:** 10.3390/ijms262311703

**Published:** 2025-12-03

**Authors:** Anna Grzesiakowska-Dul, Marek J. Kasprowicz, Olga Jarnecka, Marta Kuchta-Gładysz

**Affiliations:** 1 Department of Animals Reproduction, Anatomy and Genomics, University of Agriculture in Krakow, Mickievicza Av. 24/28, 30-059 Kraków, Poland; marta.kuchta-gladysz@urk.edu.pl; 2 Department of Soil Science and Agrophysics, University of Agriculture in Krakow, Mickievicza Av. 21, 31-120 Kraków, Poland; marek.kasprowicz@urk.edu.pl; 3 Department of Genetics, Animal Breeding and Ethology, University of Agriculture in Krakow, Mickievicza Av. 24/28, 30-059 Kraków, Poland; olga.jarnecka@urk.edu.pl

**Keywords:** genome instability, silver nanoparticles, chinchilla, sister chromatid exchange

## Abstract

The growing production and use of silver nanoparticles continues to raise questions about their consequences for human and animal health. The method of production, particle stabilization, particle size, concentration, and duration of exposure to cells can affect their reactivity and, consequently, their toxicity. This study was conducted to determine the degree of harmfulness of colloidal silver compounds, including silver nanoparticles produced via the HVAD method, to mitotic chromosomes in chinchilla’s cells. Thanks to the sister chromatid exchange (SCE) test, chromosome damage during cell division, i.e., the actual toxic effect of the tested compounds, could be assessed. For this purpose, whole peripheral blood from chinchillas was exposed in vitro to three colloidal silver compounds (unstable AgNP-HVAD, sodium citrate-stabilized silver nanoparticles—[AgNP+C], and silver nitrate) for 3, 6, and 24 h. The toxicity of these compounds was assessed at concentrations of 5, 10, and 20 µg/L and the occurrence of sister chromatid exchanges on chromosomes, resulting from double-strand DNA breaks, was analyzed. The studies revealed a notable increase in SCEs compared to the control group, suggesting the genotoxic properties of the examined AgNPs. The highest level of chromosome damage was observed following exposure to citrate-stabilized silver nanoparticles. Further research is needed to better understand the toxicological mechanisms of AgNPs produced via the HVAD method and their effects on mammalian somatic cells.

## 1. Introduction

Silver nanoparticles (AgNPs) have excellent antibacterial properties, increasing the interest in products that contain them. These particles are used in various industries, including cosmetics, textiles, medicine, and food [1,2,3]. The AgNPs in these products differ in terms of their production method, particle size, concentration, shape, and coating method. These factors determine the particles’ physicochemical properties, which may affect their toxicity to living organisms [2,4]. The ever-increasing presence of AgNPs in consumer products may lead to their bioaccumulation and release into water and soil. This could further lead to entry into the food chain [1,5,6]. Nanotechnology is currently used in agriculture to increase plant resistance and improve yields, as a growth regulator, and in nanopesticides. It is also used in animal husbandry to improve feed [3]. The food industry is investigating the applicability of AgNPs in food packaging [7]. However, such developments pose a greater risk of small particles accumulating in the environment and food chain [3]. Despite advances in nanotoxicology, there is a lack of detailed information on the potential harmful effects of nanoparticles on biological processes in the humans, animals, and plants exposed to them through domestic or industrial waste [8,9,10,11].

Due to their small size (one dimension below 100 nm), silver nanoparticles can enter the body through inhalation or skin penetration. They are then transported by the blood and lymphatic systems and can enter cells [5,12]. AgNPs easily penetrate cells, accumulate in them, block the cell cycle, induce oxidative stress, and lead to cell apoptosis. The accumulation of these processes in cells can disrupt their proper functioning, indicating a toxic effect of AgNPs. Studies on metal nanoparticles, including silver, have revealed that an important aspect resulting from the particles’ properties and interaction with cells or living organisms is their potential genotoxicity or cytotoxicity. These are associated with the potential to cause DNA damage, chromosomal changes, and mutagenic processes [11,13]. Peripheral blood lymphocytes in the body are not directly exposed to nanoparticles (or other mutagenic factors), but their constant presence in the circulatory system allows them to remain in contact with substances that have been ingested, inhaled, or adsorbed by the body, and therefore, these cells may reflect the condition of organs and tissues directly exposed to such particles or other compounds [14].

The genotoxicity of chemical or physical agents, drugs, and pesticides is assessed using cytogenetic tests. Studies on the genotoxicity of silver nanoparticles on biological material in vitro and in vivo involve tests such as the sister chromatid exchange (SCE) test [15], the micronucleus test [16,17], and the comet assay [2,18]. These tests allow for a quick, effective evaluation of the impact of AgNPs on cells and living organisms. The SCE test assesses the stability of chromosomal chromatin in cells exposed to a harmful factor. Disorders in stability are associated with a negative impact on the replication process, which may occur with AgNPs [19,20]. This may lead to changes in DNA integrity. The SCE test detects chromatin damage, including single- and double-strand DNA breaks caused by genotoxic agents and malfunctioning repair mechanisms in cells [21]. Sister chromatid exchange is indicative of the exchange of genetic material between two identical sister chromatids at homologous loci before they differentiate into two separate chromosomes during the metaphase phase of the mitotic cell. In the G2 stage, SCEs are created due to the inability to repair double-strand DNA breaks. Thus, an increased number of SCEs indicates an increased amount of DNA damage that may be caused by various factors and an increased incidence of double-strand breaks. SCE analysis is a tool for quantitatively and qualitatively assessing DNA damage caused by physical, chemical, or biological mutagenic factors [15].

Chinchillas are a species of the *Rodentia* family. Alongside rats and mice, they have gained recognition as laboratory animals due to their relatively long lifespan, ease of breeding, and gentle disposition. Their intelligence and aptitude also make them ideal subjects for behavioral studies [22,23]. Chinchillas are excellent model animals in otolaryngological research. They rarely suffer from ear diseases, and their range of hearing is similar to that of humans [22]. Cytogenetic studies on chinchillas have shown that they have 2n = 64 somatic chromosomes, comprised of 59 metacentric and 5 submetacentric chromosomes [24]. The X chromosome is the largest metacentric chromosome in the karyotype; it represents the double X chromosome type. The Y chromosome is one of the smallest metacentric chromosomes [25].

The aim of the study was to evaluate the genotoxic nature of silver nanoparticles produced via the HVAD method, as well as their ability to induce chromosomal damage in the form of sister chromatid exchanges. The study was conducted in vitro using *Chinchilla lanigera* cells to evaluate the effects of low doses, which humans and animals may be exposed to more frequently. The experimental model is a species with a well-studied karyotype and a specific level of spontaneous damage, which provided an additional reference point for analysis.

## 2. Results

### 2.1. Silver Nanoparticles

**Figure 1:**
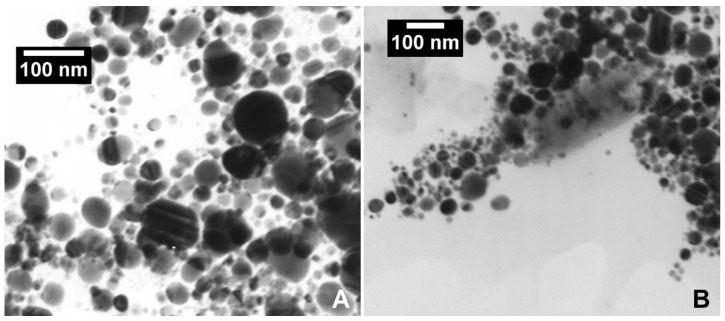
TEM image of (**A**) AgNP-HVAD nanoparticles and (**B**): AgNP+C nanoparticles.

Using the dynamic light scattering technique, the size of the nanoparticles was determined to be about 22 nm in water and about 38 nm in TSC. The electrophoretic mobility of the nanoparticles was determined using a Nano ZS Zetasizer (Malvern Instruments Ltd., Worcestershire, UK), with measurements ranging from 3 × 10^−9^–10^−5^ for zeta potential and 0.6 × 10^−9^ to 6 × 10^−6^ m for particle size. Knowing the electrophoretic mobility, the zeta potential of the particles was determined using the Henry–Smoluchowski formula. The zeta potential in colloids was estimated at −22 mV (pH 7.01) for water and −19 mV (pH 6.62) for TSC. The polydispersity index (PDI) was estimated at 0.472 for water and 0.253 for TSC. The concentration of silver nanoparticles in colloids was measured using an AAS spectrometer. TEM images of AgNP-HVAD and AgNP+C nanoparticles are shown in Figure 1A,B.

### 2.2. Sister Chromatid Exchange

Sister chromatid exchanges were counted from well-spaced, differentially stained metaphase plates. Examples of these plates with marked SCEs are shown in Supplementary Figure S1. The average number of SCEs per cell in the control sample was 1.57 ± 1.10. A higher frequency of chromosomal damage, as observed by SCEs, was found in all experimental groups compared to the control. The differences in the mean number of SCEs per cell between the control and all test variants (solution, dose, and time) were highly significant (*p* < 0.001). Further analysis assessed whether the number of SCEs depended on the colloidal silver solution, its concentration, exposure time, or the interaction between these factors (Table 1).

The analysis revealed that the number of sister chromatid exchanges was not influenced by individual factors, such as solution type, concentration, or time. The interaction between solution and time was significant relative to the control; that is, the number of SCEs varied depending on the duration of exposure to individual solutions (Table 1). Samples exposed to AgNP-HVAD exhibited fewer induced SCEs/cell after 24 h (4.83 ± 2.62) than after 6 h (5.90 ± 3.01) or 3 h (5.59 ± 2.85). In the experimental groups exposed to citrate-stabilized silver nanoparticles, no differences in the number of induced SCEs per cell were observed at different time intervals. Silver nitrate exposure resulted in fewer SCEs after 6 h (5.00 ± 2.09) than after 3 h (5.55 ± 2.76) or 24 h (5.74 ± 2.54) (Table 1).

The average number of SCEs in chinchilla blood cells was found to vary depending on the concentration of the colloidal silver solution. In experimental trials using AgNP-HVAD and AgNO_3_ solutions, the average number of SCEs remained consistent across different concentrations (5/10/20 μg/L). However, in the case of AgNP+C, a significantly lower average SCE count was observed at 20 μg/L (4.62 ± 2.02) compared to 5 μg/L (6.38 ± 3.33) and 10 μg/L (6.00 ± 2.94) (see Table 1).

The exposure time of silver solutions revealed a variation in the average number of SCEs, depending on the concentration. It also depended on the type of solution being tested (Table 1). At the lowest tested concentration, 5 μg/L, a lower frequency of SCEs was observed after 6 h of exposure (3.80 ± 1.75 to 5.36 ± 3.36) than after 3 h (5.84 ± 2.69 to 7.32 ± 3.28) or 24 h (5.73 ± 2.22 to 6.46 ± 3.34). After exposure to 10 and 20 μg/L, no significant differences were observed, regardless of the duration of exposure. No clear, recurring trends were found among the groups in terms of solution type, concentration, or exposure time.

The citrate-stabilized silver nanoparticles (AgNP+C) exhibited the highest level of genotoxicity, as evidenced by the higher average number of sister chromatid exchanges. This solution induced significantly more damage at the lowest concentration (5 μg/L) compared to AgNP-HVAD and AgNO_3_. At higher concentrations (10 and 20 μg/L), the level of damage induced by all tested solutions was similar. Silver nitrate exhibited significantly stronger genotoxic properties than the unstable AgNP-HVAD. The latter solution had a stronger effect than AgNO_3_ at the lowest concentration over a longer exposure period (6 and 24 h).

For the control sample, the frequency of SCEs at various locations on the chromosome was determined as follows: terminal SCE 0.96 ± 0.87; centromeric SCE 0.60 ± 0.76; and interstitial SCE 0.01 ± 0.12. The high variability of these measurements may be due to the low frequency of SCEs in the interstitial and centromeric locations of the cells from the control group.

Significant differences in the frequency of exchanges were found for most of the tested variants when comparing the terminal, centromeric, and interstitial regions. The exceptions were interstitial SCEs for 5 μg/L of AgNP-HVAD, after 3 h (0.21 ± 0.41), after 6 h (0.22 ± 0.42), and with 10 μg/L after 6 h (0.09 ± 0.29). No SCEs were observed in the interstitial position with 20 μg/L of AgNP-HVAD after 6 h (Table 2, Table 3 and Table 4).

Statistically significant differences were found regarding average SCE, terminal, and centromeric damage in almost all concentration and time variants of the samples treated with AgNP+C solution versus the control. However, no significant differences were found in the 10 μg/L, 6 h sample (mean SCE: 6.57 ± 2.34; terminal SCE: 4.50 ± 1.70; centromeric SCE: 2.00 ± 0.88). Significant differences compared to the control were found in interstitial SCE in the 5 μg/L, 3 h (0.11 ± 0.31) and 5 μg/L, 24 h (0.18 ± 0.39) variants, as well as in the 20 μg/L, 3 h (0.16 ± 0.43) and 20 μg/L, 6 h (0.12 ± 0.33) variants (Table 2, Table 3 and Table 4).

The reference solution for testing the toxicity of the silver nanoparticles was silver nitrate. Statistically significant differences in the mean SCE, terminal, and centromeric values were found at all concentrations and time points compared to the control. Significant differences in interstitial SCE count were observed in the following samples: 5 μg/L, 3 h (0.11 ± 0.32) 5 μg/L, 24 h (0.12 ± 0.33), 10 μg/L, 3 h (0.46 ± 0.58), 10 μg/L, 6 h (0.33 ± 0.60), 10 μg/L, 24 h (0.31 ± 0.54), and 20 μg/L, 24 h (0.15 ± 0.46). No interstitial exchanges were identified in the AgNO_3_ groups: 5 μg/L, 6 h; 20 μg/L, 3 h; and 20 μg/L, 6 h (Table 2, Table 3 and Table 4).

The analysis of sister chromatid exchange frequencies in terminal positions revealed higher values in the experimental samples than in the controls (0.96 ± 0.87), confirming silver compounds’ ability to induce chromosomal damage. Overall, no significant effect of individual factors, such as solution type, concentration, or exposure time, was observed on terminal SCE frequency (Table 2).

Significant differences only occurred in select combinations of concentration and exposure time. At a concentration of 5 μg/L after 6 h of exposure, the number of SCEs was significantly lower in the AgNO_3_-treated group than in the silver nanoparticle solution groups.

The greatest variation was observed at a concentration of 10 μg/L after 6 h: the AgNP+C solution induced the highest number of exchanges, while AgNO_3_ induced the lowest. After 24 h of exposure at the same concentration (10 μg/L), the number of SCEs was lower in the AgNP+C group than in the others. A similar trend was observed for 20 μg/L after 24 h: the AgNP+C solution caused significantly fewer SCEs than the AgNP-HVAD and AgNO_3_ solutions.

The results indicate that the intensity of sister chromatid exchanges at the ends of chromosomes depends mainly on the interaction between the solution type and exposure time. The highest values were most often observed after 6 h of exposure to silver nanoparticles (Table 2).

Sister chromatid exchanges at the centromeric position exhibited a similar incidence among the experimental groups, depending on exposure time or silver solution concentration; they differed significantly from the control group (Table 3). No differences were found when analyzing the effect of exposure time of individual solutions, and a similar level of SCEs was observed in chinchilla blood cells. The frequency range of these exchanges was 2.09 to 2.45 SCE/cell. The lowest value for exchanges induced in this chromosomal region was observed after 6 h of exposure to AgNO_3_, while the highest level was observed after 3 h of exposure to AgNP+C. Chinchilla chromosome sensitivity in the centromeric region depended on the concentration and type of AgNP; it ranged from 1.97 (20 μg/L of AgNP-HVAD) AgNP+C to 2.73 (5 μg/L of AgNP+C). In contrast, an extremely high number of SCEs in this region was found with 5 μg/L of AgNP-HVAD: 6.44 SCEs/cell.

Interstitial sister chromatid exchanges were the least frequent type of damage analyzed. Their number was significantly higher in all experimental groups compared to the control group (*p* < 0.001), which had a mean value of 0.01 ± 0.12 SCEs/cell. No significant differences were found between solutions or exposure times. The mean values remained low and similar (0.05–0.23 SCEs/cell). However, significantly higher values (*p* < 0.05) compared to the other groups were recorded in the group treated with a 10 μg/L silver nitrate solution after 3 and 6 h of exposure (Table 4).

## 3. Discussion

The development and widespread use of nanomaterials has led to a constant increase in the threat to human and animal health, as well as to the environment. Nanotoxicology is the study of the impact of new materials on organisms, their toxicity, and their ability to interact with and affect organisms, systems, cells, proteins, hormones, and other factors. According to some scientists, this field is also referred to as nanogenotoxicology [3,13,26]. Due to their small size, surface reactivity, and ability to transfer and penetrate active substances, nanomaterials often exhibit cytotoxic and/or genotoxic effects. The genotoxic effect of such agents is associated with their potential interaction with cellular DNA, which can result in damage to DNA strands [27]. Silver nanoparticles have been proven to cause DNA damage and chromosomal aberrations, leading to cell cycle arrest [19,28,29].

The particles small size (less than 10 nm) allows them to penetrate cells and enter the cell nucleus via various routes, such as nuclear pores or endocytosis. Once inside the nucleus, the nanomaterial can interact directly with DNA molecules and disrupt cell replication during the interphase stage and mitosis. It can also disrupt the action of the mitotic spindle and centrioles, which affects the segregation of chromosomes into cells and ultimately causes a mutagenic effect. Other toxicity mechanisms involve the induction of reactive oxygen species (ROS) or the release of ions from the particle’s surface that interact with cellular proteins and DNA [11,27].

One approach to assessing the toxicity of new substances and materials is to evaluate a factor’s ability to generate DNA damage and cause chromosomal aberrations. The sister chromatid exchange (SCE) assay is a cytogenetic assay that assesses damage at the chromosomal level [13,29]. SCE damage occurs spontaneously during cell proliferation and is considered a symptom of genome damage. The SCE test measures genotoxic and mutagenic effects in the cytogenetic response to chemical exposure in a dose–response relationship. Sister chromatid exchanges are one of the genotoxic endpoints that reflect DNA damage or exposure to a given factor in biological dosimetry analysis [15]. This method is also used to assess the genotoxicity of nanomaterials [13,20], and some authors observe these changes in the general chromosome aberration test [30].

In vivo and in vitro studies have demonstrated that silver nanoparticles induce chromosome and DNA damage, in both mammalian and fish cells [3,29,31]. Nanoparticles can interact directly with DNA when they penetrate cells. However, DNA damage can also arise through indirect mechanisms without entering the cell. In these cases, nanomaterials interact with other cellular proteins, such as those involved in the cell division process, rather than with DNA molecules directly. Additionally, nanomaterials can trigger cellular responses that lead to genotoxicity, such as oxidative stress, inflammation, and abnormal signaling. The sister chromatid exchange analysis focuses on these effects to measure genotoxicity and mutagenicity [32]. Mohamed [31] confirmed the dose-dependent genotoxicity of a silver nanoparticle solution in terms of chromosome and DNA damage. Ghosh et al. [8] demonstrated the genotoxicity of AgNPs in vitro on mouse bone marrow cells. They found that the percentage of abnormal cells was significantly higher after AgNP induction compared to the control. The most frequently observed damage was chromatid breaks. These observations are consistent with our study, in which there was also a higher incidence of damage in all experimental groups exposed to silver compounds compared to the control group. The SCE test is based on detecting chromatid breaks within chromosomes. It confirms the observations of Ghosh et al. [8], as well as the clastogenic effect of silver nanoparticles.

A study by Hackenberg et al. [18] reported the impact of AgNP dosage on the frequency of chromosomal damage. The study demonstrated that AgNP concentrations of 0.1 μg/L or higher (1 μg/L and 10 μg/L) caused more chromosomal damage than the control group. The main structural aberrations observed in these tests were deletions and chromatid exchanges. Our studies confirmed the higher frequency of chromatid exchanges using the SCE test for each silver solution, including silver nanoparticles, at a concentration of 5 μg/L or higher, with a significant rise at 10 μg/L. Additionally, Mecwan et al. [33] analyzed chromosomal aberrations in human blood and found significantly higher and dose-dependent damage in cells treated with AgNPs (green particle synthesis) compared to the untreated group, except at the lowest concentration they used (350 μg/L).

Güzel et al. [11] conducted a comparative analysis of the effects of silver nanoparticles and various silver halides used medically as antimicrobials on human peripheral blood cells in vitro. They used concentrations of 0.5; 1; 5; and 10 mM and 24 and 48 h of exposure. In the chromosome aberration test, which, like the SCE test, detects breaks in chromosomes, the cytotoxic effect of ionic silver was demonstrated, causing more breaks after 24 h of exposure at the two lowest doses. After 48 h, the authors found no significant differences in damage compared to the control. However, the effect of AgNP was determined to be significant at a dose of 0.5 mM after 24 h, as a significant increase in structural damage to chromosomes was observed. At higher doses of AgNP, no significance was found. In our studies, a higher level of damage induced by silver ions was found only at the lowest dose (5 μg/L) after 3 h of exposure. Longer exposure to and higher concentrations of AgNO_3_ and AgNP resulted in equalization of SCE levels, with those caused by AgNP predominating.

Previous studies on these colloidal silver compounds [34] using the comet assay have demonstrated the importance of time, concentration, and their interaction for AgNP-HVAD and AgNP+C in terms of the amount of changes they induce in chromatin structures within chinchilla nuclei. The factor that most strongly disrupted DNA integrity in the interphase nucleus was the sodium citrate-stabilized AgNP solution (AgNP+C). In this study, this effect was confirmed at the chromosomal level for two concentrations (5 and 10 μg/L). A comparison of the two studies reveals that the duration of exposure of the silver compound to the cell cycle stage and the form of genome organization are critical factors that can differentiate their impact on nuclear structures. AgNP-HVAD exhibited toxicity comparable to AgNO_3_ at the chromosomal level; however, it was a weaker inducer of nuclear chromatin in the interphase nucleus than nitrate. The results of both studies are consistent with the observations collected by Rija et al. [20].

Sister chromatid exchanges can also be observed through spontaneous damage to living organisms’ cells. Kuchta-Gładysz et al. [24] assessed this non-induced level of SCE in chinchillas to be an average of 3.40 ± 0.98 SCEs/cell. The highest frequency of changes occurred in the proximal position (59%), while the distal position showed a lower frequency (39%). In the most recent studies, the SCE frequency in the control group, which can be equated to spontaneous changes, was less than half. This difference may result from the different sample sizes of the study groups in these two experiments. Additionally, this value may indicate that the animals were in good health and the analysis was performed correctly without inducing additional damage that could have distorted the results. The analyzed silver solutions showed more frequent SCE induction in the distal-terminal region, with an average of more than three exchanges per cell. In the proximal region, the average was two exchanges per cell. The proximal region of chinchilla chromosomes contains constitutive heterochromatin. In contrast, the terminal and distal regions may be more susceptible to damage, especially from oxidative stress, than other regions [21].

## 4. Materials and Methods

### 4.1. Material—Animals

The material for the research was whole peripheral blood of 12 chinchillas (*Chinchilla lanigera*) of the Standard strain. Approximately 2–3 mL of whole blood was collected post mortem in sterile sample tubes with lithium heparin (FL Medical, Torreglia, Italy). The animals were of the same age (1 year). The chinchillas were kept in accordance with the European Convention for the Protection of Vertebrate Animals, complying with the conditions stipulated in the Act of 29 June 2007, presently in force in Poland. Ethics committee approval is not required for the use of biological material collected post mortem from animals previously bred on livestock farms. At the same time, we emphasize that no animal was sacrificed for the purposes of this experiment.

### 4.2. Material—Silver Nanoparticles

The silver nanoparticles were produced using a high-voltage discharge in an electric arc [35,36]. Two electrodes made of 99.9% pure silver and 10 mm and 5 mm in diameter were placed in a reactor with a capacity of ~1 × 10^−5^ m^3^ and immersed in double-distilled water (conductivity—(6–10) × 10^−6^ Si) or 3.3 µM water solutions of tri-sodium citrate dihydrate (TSC) (pure, POCH, Gliwice, Poland). The electrical voltage between the electrodes was 20 kV. The distance between the electrodes was 2.5 × 10^−4^ m. The transmission electron microscopy (TEM) images were taken using a JEOL JEM 100SX transmission electron microscope (JEOL, Tokyo, Japan).

### 4.3. Methods—Cell Exposure to Silver Nanoparticles

Before establishing a cell culture from blood for the SCE test, the material was exposed to three silver solutions: silver nanoparticles in distilled water (AgNP-HVAD), silver nanoparticles stabilized with a sodium tris (citrate) solution (AgNP+C), and silver nitrate (AgNO_3_). The cells were exposed for 3, 6, or 24 h. Additionally, the effects of three concentrations (5, 10, and 20 μg/L) of the tested solutions were examined during the cells’ exposure to the silver solution. Whole blood exposure was conducted in sterile Eppendorf tubes after the tested silver solutions and physiological saline (PBS, Sigma-Aldrich, Poznań, Poland) were sterilized. PBS was added to the blood sample to serve as a negative control and to appropriately dilute the sample, analogous to the experimental samples. A 1:1 mixture of whole peripheral blood and an appropriate silver solution at a concentration of 5, 10, or 20 µg/L was prepared in Eppendorf tubes (FL Medical, Torreglia, Italy) for the experimental samples, while the negative control contained PBS. All procedures were performed under sterile conditions.

### 4.4. Methods—Cell Cultures for Cytogenetic Assay

Peripheral blood lymphocytes were cultured in LymphoGrow medium (Cytogen, Zgierz, Poland) at a constant humidity of 37.5 °C for 72 h. The SCE test was performed according to the procedure described by Di Berardino et al. [37]. After 24 h of in vitro culturing, 1 μg/mL of BrdU (Sigma-Aldrich, Poznań, Poland) was added to the cells as an indicator for the SCE test. To obtain metaphase chromosomes, 1 μg/mL of colchicine (Sigma-Aldrich, Poznań, Poland) was added after 70 h of culturing. The cell culture was established and maintained under sterile conditions to minimize contamination of the cells and the sample. At the conclusion of the culture period, the cells were hypotonicized using a KCl solution (Sigma-Aldrich, Poznań, Poland) and subsequently fixed in Carnoy’s solution (a 3:1 mixture of methanol and acetic acid; POCH, Gliwice, Poland).

### 4.5. Methods—Sister Chromatid Exchange Assay

Cell suspensions were spotted onto slides and differentially stained using the fluorescence plus Giemsa technique according to Kihlman and Kronborg [38]. The chromosome preparations were treated with RNase and incubated in a Hoechst solution in a 0.5× SSC buffer (all from Sigma-Aldrich, Poznań, Poland). The slide was then exposed to UV light and incubated overnight in the dark at 4 °C. Then, the microscopic slides were re-irradiated with UV light, incubated in 0.5× SSC buffer at 58 °C, and stained with 3% Giemsa in Sorensen’s buffer (all from Sigma-Aldrich, Poznań, Poland). Twenty complete and differentially stained metaphase plates were analyzed for each sample to determine the number of SCEs per cell and their location on the chromosomes.

### 4.6. Microscopic Analysis

Microscopic analysis and photographic documentation were performed using a Carl Zeiss Jena-Jenaval microscope (Carl Zeiss, Düsseldorf, Germany) coupled with a Nikon DS-Fi1 digital camera (Nikon, Tokyo, Japan) and NIS-Elements image analysis software ver. F2.31 (Nikon, Tokyo, Japan).

### 4.7. Statistical Analysis

The data of 12 animals were used in the statistical analysis. The normality of data distribution was verified using the Shapiro–Wilk test. Since the results did not follow a normal distribution, non-parametric tests were applied. In the first stage, the cytotoxic effect of the tested silver compounds was evaluated based on the mean number of sister chromatid exchanges (SCEs) per cell, depending on the solution type (AgNP-HVAD, AgNP+C and AgNO_3_), concentration (5, 10, and 20 μg/L), and exposure time (3, 6, and 24 h). Subsequently, the occurrence of SCEs located in terminal, centromeric, and interstitial regions of chromosomes was analyzed in relation to the same factors. Comparisons between the above-mentioned chromatid features were made in the control group and in the treated group using the Kruskal–Wallis test, followed by the Dwass–Steel–Critchlow–Fligner test for post hoc pairwise comparisons. Statistical significance was set at *p* < 0.05. All statistical analyses were carried out using the SAS software package 13.2 (SAS Institute Inc., Cary, NC, USA) [39].

## 5. Conclusions

The higher number of SCEs observed in cells exposed to colloidal silver solutions, as compared to the control group, confirms the genotoxic and clastogenic effects of AgNPs obtained via the HVAD method. Studies have shown that low concentrations of AgNPs, which we may be exposed to constantly, cause changes in chromosome structure. After 24 h of exposure, the accumulated changes did not cause cell death or rapid destabilization of the chinchilla genome. However, it should be noted that structural changes and damage to nuclear chromatin that accumulate in cells over time may pose a threat to human and animal health.

## Figures and Tables

**Table 1:** Cytotoxic effect of silver compounds determined by the average number of SCEs, depending on the solution, time, and concentration.

Number of SCEs					
Solutions	Time [h]	Concentration [μg/L]			Mean Values for the Solution
		5	10	20	
AgNP-HVAD	3	5.84 ± 2.69	5.10 ± 3.19	5.84 ± 2.67	5.59 ± 2.85
	6	4.84 ± 2.75	7.24 ± 4.22	5.62 ± 2.06	5.90 ± 3.01
	24	6.00 ± 2.96	4.36 ± 2.37	4.12 ± 2.52	4.83 ± 2.62
	mean	5.56 ± 2.73	5.57 ± 3.26	5.19 ± 2.42	-
AgNP+C	3	7.32 ± 3.28	5.39 ± 3.68	4.77 ± 2.98	5.83 ± 3.31
	6	5.36 ± 3.36	6.57 ± 2.34	5.71 ± 1.96 ^b^	5.88 ± 2.55
	24	6.46 ± 3.34 ^a^	6.02 ± 2.80	3.39 ± 1.13 ^ab^	5.29 ± 2.42
	mean	6.38 ± 3.33	6.00 ± 2.94	4.62 ± 2.02	-
AgNO_3_	3	6.21 ± 3.25 ^a^	5.54 ± 2.95	4.91 ± 2.07	5.55 ± 2.76
	6	3.80 ± 1.75 ^ab^	6.21 ± 2.33	5.00 ± 2.18	5.00 ± 2.09
	24	5.73 ± 2.22 ^b^	5.40 ± 2.13	6.11 ± 3.27	5.74 ± 2.54
	mean	5.25 ± 2.41	5.72 ± 2.47	5.34 ± 2.51	-
Control	1.57 ± 1.10				

Mean ± standard deviation; means marked with the same letters ^a,b^ differ significantly (*p* < 0.05).

**Table 2:** Appearance of terminal SCEs depending on solution, time, and concentration.

Terminal SCE					
Solutions	Time [h]	Concentration [μg/L]			Mean Values for the Solution
		5	10	20	
AgNP-HVAD	3	3.45 ± 1.58	2.67 ± 2.14	3.40 ± 2.42	3.17 ± 2.05
	6	3.02 ± 1.89	4.35 ± 3.17	3.69 ± 1.70	3.69 ± 2.25
	24	3.03 ± 1.95	2.46 ± 1.74	1.94 ± 1.78	2.48 ± 1.82
	mean	3.17 ± 1.81	3.16 ± 2.35	3.01 ± 1.97	-
AgNP+C	3	3.94 ± 1.74	2.89 ± 2.18	3.00 ± 1.69	3.28 ± 1.87
	6	3.19 ± 2.01	4.50 ± 1.70	3.18 ± 1.53	3.62 ± 1.75
	24	3.49 ± 1.56	3.73 ± 2.45	1.44 ± 1.18	2.89 ± 1.73
	mean	3.54 ± 1.77	3.71 ± 2.11	2.54 ±1.47	-
AgNO_3_	3	3.87 ± 2.17	2.48 ± 1.88	3.36 ± 1.21	3.24 ± 1.75
	6	2.13 ± 1.01	1.89 ± 1.54	2.73 ± 1.16	2.25 ± 1.24
	24	3.49 ± 1.53	2.85 ± 1.63	3.15 ± 1.85	3.16 ± 1.67
	mean	3.16 ± 1.57	2.41 ± 1.68	3.08 ± 1.41	-
Control	0.96 ± 0.87				

Mean ± standard deviation.

**Table 3:** Occurrence of centromeric SCEs depending on solution, time, and concentration.

Centromere SCE					
Solutions	Time [h]	Concentration [μg/L]			Mean Values for the Solution
		5	10	20	
AgNP-HVAD	3	2.18 ± 1.78	2.27 ± 1.81	2.08 ± 1.04	2.18 ± 1.54
	6	1.63 ± 1.34	2.80 ± 1.63	1.94 ± 0.68	2.12 ± 1.22
	24	2.63 ± 1.78	1.84 ± 1.54	1.88 ± 1.32	2.12 ± 1.55
	mean	6.44 ± 1.63	2.28 ± 1.66	1.97 ± 1.01	-
AgNP+C	3	3.28 ± 2.57	2.46 ± 2.18	1.61 ± 1.56	2.45 ± 2.10
	6	2.13 ± 1.89	2.00 ± 0.88	2.41 ± 1.16	2.18 ± 1.31
	24	2.79 ± 2.30	2.29 ± 0.93	1.89 ± 1.17	2.32 ± 1.47
	mean	2.73 ± 2.25	2.25 ± 1.33	1.97 ± 1.30	-
AgNO_3_	3	2.23 ± 1.73	2.62 ± 1.78	1.55 ± 1.04	2.13 ± 1.52
	6	1.67 ± 1.18	2.33 ± 1.61	2.27 ± 1.45	2.09 ± 1.41
	24	2.10 ± 1.24	2.26 ± 1.56	2.82 ± 2.02	2.39 ± 1.61
	mean	2.00 ± 1.38	2.40 ± 1.65	2.21 ± 1.50	-
Control	0.60 ± 0.76				

Mean ± standard deviation.

**Table 4:** Occurrence of interstitial SCEs depending on solution, time, and concentration.

Interstitial SCE					
Solutions	Time [h]	Concentration [μg/L]			Mean Values for the Solution
		5	10	20	
AgNP-HVAD	3	0.21 ± 0.41	0.18 ± 0.43	0.24 ± 0.52	0.21 ± 0.45
	6	0.22 ± 0.42	0.09 ± 0.29	0.00 ± 0.00	0.10 ± 0.24
	24	0.28 ± 0.61	0.10 ± 0.30	0.29 ± 0.59	0.22 ± 0.50
	mean	0.23 ± 0.48	0.12 ± 0.34	0.18 ± 0.37	-
AgNP+C	3	0.11 ± 0.31	0.04 ± 0.19	0.16 ± 0.43	0.10 ± 0.31
	6	0.03 ± 0.18	0.07 ± 0.27	0.12 ± 0.33	0.07 ± 0.26
	24	0.18 ± 0.39	0.00 ± 0.00	0.06 ± 0.23	0.08 ± 0.21
	mean	0.11 ± 0.29	0.04 ± 0.15	0.11 ± 0.33	-
AgNO_3_	3	0.11 ± 0.32	0.46 ± 0.58	0.00 ± 0.00	0.19 ± 0.30
	6	0.00 ± 0.00	0.33 ± 0.60	0.00 ± 0.00	0.11 ± 0.60
	24	0.12 ± 0.33	0.31 ± 0.54	0.15 ± 0.46	0.19 ± 0.44
	mean	0.08 ± 0.22	0.37 ± 0.41	0.05 ± 0.15	-
Control	0.01 ± 0.12				

Mean ± standard deviation.

## Data Availability

The original contributions presented in this study are included in the article/Supplementary Material. Further inquiries can be directed to the corresponding author.

## Supplementary Materials

The following supporting information can be downloaded at https://www.mdpi.com/article/10.3390/ijms262311703/s1.
