# Supplementary material for: Genotoxicity Assessment of Silver Nanoparticles Produced via HVAD: Examination of Sister Chromatid Exchanges in Chinchilla lanigera Blood Lymphocytes In Vitro
Source: Int J Mol Sci. 2025 Dec 3;26(23):11703. doi: 10.3390/ijms262311703 (PMC12692570; doi:10.3390/ijms262311703)
Supplement: Supplementary file 1 [file ijms-26-11703-s001.zip › ijms-3971987-supplementary.pdf]

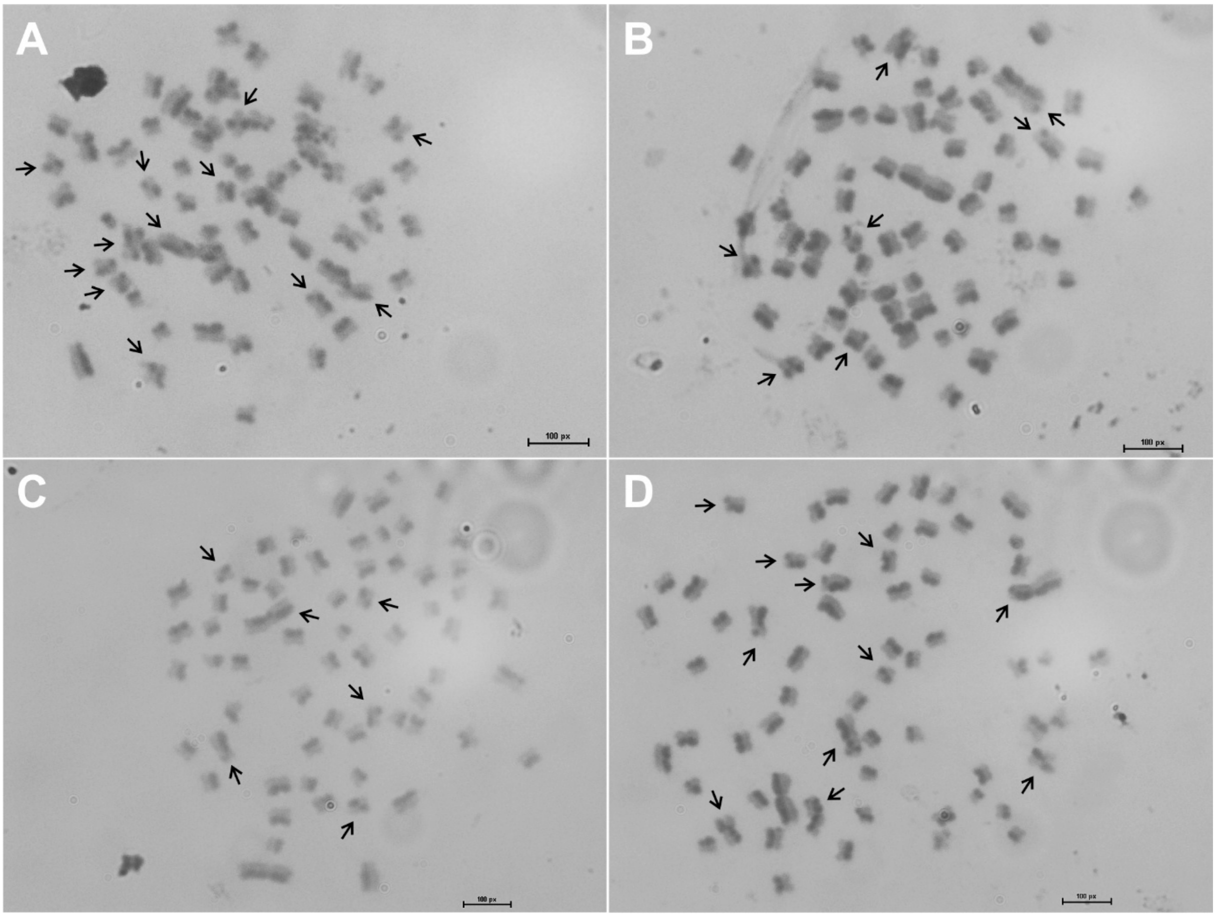

Figure S1: Metaphase plates with chinchilla chromosomes (2n = 64) after three hours of exposure to the tested silver compounds in 5 μg/L concentration: A. AgNP-HVAD, B. $AgNO_3$, C. control, and D. AgNP+C. The arrows indicate sister chromatid exchanges on chromosomes. Scale: 10 μm.
